# Clinical and magnetic resonance imaging characterization of cervical spondylomyelopathy in juvenile dogs

**DOI:** 10.1111/jvim.15602

**Published:** 2019-08-30

**Authors:** Marília de Albuquerque Bonelli, Ronaldo C. da Costa

**Affiliations:** ^1^ Department of Veterinary Clinical Sciences, College of Veterinary Medicine The Ohio State University, 601 Vernon Tharp St. Columbus Ohio

**Keywords:** cervical spine, diagnostic imaging, retrospective study, wobbler syndrome

## Abstract

**Background:**

Cervical spondylomyelopathy (CSM) occurs because of compression of the cervical spinal cord, nerve roots, or both, usually affecting young adult to older large and giant breed dogs. Juvenile dogs are affected infrequently.

**Objective:**

To describe clinical and magnetic resonance imaging (MRI) findings in juvenile dogs (≤ 12 months) with cervical spondylomyelopathy.

**Animals:**

Twenty CSM‐affected juvenile dogs.

**Methods:**

Medical and imaging records for juvenile dogs with CSM were reviewed. History and neurologic examination findings were obtained, including follow‐up data. The MRI studies were reviewed for cause and site of spinal cord compression, intervertebral disk protrusion or degeneration, articular process degenerative changes, intervertebral foraminal stenosis, and spinal cord signal changes.

**Results:**

Mean (median) age at the time of diagnosis was 9.4 (10) months. There were 16 giant breed dogs. Eighteen dogs had a chronic presentation, 18/20 had proprioceptive ataxia, and 9/20 had cervical pain. On MRI, the principal spinal cord compression occurred at C5‐C6, C6‐C7, or both in most dogs; 12/20 dogs had ≥2 sites of spinal cord compression. The cause of compression was articular process proliferation in 8/20 dogs and disk protrusion in 2/20 dogs. Intervertebral disk degeneration was seen in 9/20 dogs. Follow‐up was obtained for 12/20 dogs: 10/12 were managed medically and 2/12 surgically.

**Conclusions and Clinical Importance:**

Cervical spondylomyelopathy in juvenile dogs was characterized mostly by osseous‐associated spinal cord compression and multiple compressive sites. Almost half of the dogs had intervertebral disk degeneration. Intervertebral disk protrusion was seen in both giant and large breed dogs.

**Abbreviations**
CSF: cerebrospinal fluid
CSM: cervical spondylomyelopathy
CT: computed tomography
DA‐CSM: disk‐associated cervical spondylomyelopathy
MRI: magnetic resonance imaging
OA‐CSM: osseous‐associated cervical spondylomyelopathy
T1W: T1‐weighted
T2W: T2‐weighted
TP: total protein

## 1 INTRODUCTION

Cervical spondylomyelopathy (CSM), or wobbler syndrome, is characterized by compression of the spinal cord, nerve roots, or both in the cervical vertebral column, usually affecting large and giant breed dogs.^1^, ^2^ The most commonly affected giant breed is the Great Dane,^3^, ^4^, ^5^ whereas the most commonly affected large breed is the Doberman Pinscher.^6^, ^7^

The pathogenesis of CSM is not entirely understood, but it is thought to involve dynamic and static factors. These factors also are influenced by vertebral canal stenosis, degree of spinal cord compression, movement‐associated compressions, shape of the vertebral canal and vertebral bodies, as well as molecular mechanisms.^1^, ^2^

Large breed dogs usually develop CSM after 3 years of age because of disk‐associated CSM (DA‐CSM) with compression secondary to protrusion of intervertebral disks, and often present with neurologic signs at mean and median ages of 7 and 7.1 years, respectively.^8^, ^9^ Giant breed dogs more commonly develop osseous‐associated CSM (OA‐CSM), which usually results from osseous proliferation of the articular processes leading to spinal cord, nerve root compression, or both around a mean age of 3.8 years and median age of 2.5 years, although they can develop signs at a younger age.^3^, ^5^, ^10^, ^11^

One of the difficulties in trying to understand the course of CSM in a population of young dogs is the small number of CSM‐related studies that include juvenile dogs.^10^, ^11^, ^12^ Another problem is that the exact number of juvenile dogs often is not given in these reports; therefore, it is difficult to know which clinical or imaging findings pertain to young dogs. Of all papers reviewed from the last 45 years that reported juvenile dogs in their population,^3^, ^4^, ^6^, ^10^, ^13^, ^14^, ^15^, ^16^, ^17^, ^18^, ^19^, ^20^, ^21^, ^22^, ^23^, ^24^, ^25^, ^26^ only 7 specified how many dogs were ≤ 1 year.^13^, ^15^, ^17^, ^18^, ^21^, ^22^, ^23^ The juvenile form of CSM has not been thoroughly investigated in any study.

Our objective was to characterize the clinical and magnetic resonance imaging (MRI) findings in juvenile dogs (up to 12 months of age) with CSM.

## 2 MATERIALS AND METHODS

Medical and imaging records were searched from 2007 to 2018 for dogs ≤12 months of age with a confirmed diagnosis of CSM based on clinical signs and MRI findings. Search terms included: “cervical spondylomyelopathy,” “CSM,” and “wobbler.” Inclusion criteria for the study therefore were the diagnosis on MRI of CSM in dogs ≤12 months of age at the time of MRI examination and medical records with high‐field MRI available for review. Exclusion criteria were age > 12 months or incomplete medical records.

Signalment, history, ancillary diagnostic testing, and neurologic examination findings were obtained from patient records, focusing on age, duration of clinical signs (acute when <1 week, chronic when ≥1 week before diagnosis), presence of ataxia, paresis or both, presence of cervical hyperesthesia, as well as treatment option (conservative or surgical) and follow‐up.

The neurologic status of the dogs was graded using a grading system from 1 to 5 (adapted from a previously published system),^27^ as follows: grade 1, cervical hyperesthesia only; grade 2, mild pelvic limb ataxia or paresis with or without thoracic limb involvement; grade 3, moderate pelvic limb ataxia or paresis with or without thoracic limb involvement; grade 4, marked pelvic limb ataxia or paresis with thoracic limb involvement; and grade 5, tetraparesis or inability to stand or walk without assistance.

Magnetic resonance imaging of the cervical vertebral column was reviewed by a board‐certified neurologist (RCdC). The magnetic resonance images were acquired using a high‐field (3.0 T or 1.5 T) MRI scanner, with variable protocols, but included at least sagittal T1‐weighted (T1W) and T2‐weighted (T2W) views of the cervical vertebral column from C2 to T1 and transverse T1W and T2W views of at least the sites of spinal cord compression. A diagnosis of CSM was confirmed by MRI when ≥1 sites of compression of the spinal cord, nerve roots, or both were observed because of intervertebral disk protrusion, ligamentum flavum hypertrophy, osseous proliferation of the articular processes, or thickening of the dorsal lamina, as well as the presence of absolute or relative stenosis of the vertebral canal.^1^ The following information was recorded from the MRIs: cause of spinal cord compression, main compression site, direction of compression (ventral, lateral, dorsal, or any combination thereof), intervertebral disk degeneration, presence of synovial joint fluid at the articular process joint (normal, decreased, absent), regularity of the articular surface and presence of subchondral bone sclerosis at the articular process joint (smooth without signs of subchondral bone sclerosis, smooth with evidence of subchondral bone sclerosis, irregular with subchondral bone sclerosis), intervertebral foraminal stenosis, and spinal cord signal changes on T1‐ and T2‐weighted images. The criteria to determine these changes followed previous studies on MRI and CSM.^4^, ^8^, ^10^, ^11^, ^28^ The cause of spinal cord compression was classified as intervertebral disk protrusion, proliferation of the articular processes, hypertrophy of the ligamentum flavum, thickening of the dorsal lamina, or any combination of these. Spinal cord compression was classified as mild (compression <25% of the cross‐sectional area of the noncompressed spinal cord immediately cranial and caudal to the compression), moderate (25‐50%), or severe (> 50%).^8^, ^11^ The main compression site was chosen as the site with the greatest reduction in cross‐sectional area and T2‐weighted hyperintensity (when present).

## 3 RESULTS

Twenty dogs met the inclusion criteria: 13 Great Danes, 3 Mastiffs, 2 Dobermans, 1 Bloodhound, and 1 German Shorthaired Pointer. Based on breed, 16 dogs were classified as giant breeds and 4 as large breeds. Five of these dogs had been included in previous prospective studies carried out by the senior author (Marília de Albuquerque Bonelli).^11^, ^12^

Mean and median ages at the time of diagnosis were 9.4 and 10 months (range 3.7‐12 months), respectively. There were 14 males and 6 females. Eighteen dogs had a chronic history with a mean 7.3 week duration of clinical signs (median, 4.5 weeks; range, 1.5‐32 weeks), whereas 2 dogs had an acute presentation.

Eleven (11/20) dogs presented with tetraparesis, 18/20 with proprioceptive ataxia, and 9/20 with cervical pain. All dogs with tetraparesis also had ataxia. Neurologic grade was classified as grade 1 in 2/20 dogs, grade 2 in 7/20 dogs, grade 3 in 5/20 dogs, and grade 4 in 6/20 dogs.

The findings of conscious proprioceptive testing were as follows: decreased to absent in all limbs in 15/20 dogs (worse in the pelvic limbs in 5/15 and worse in the thoracic limbs in 3/15), decreased only in the pelvic limbs (worse on left) in 2 dogs, decreased only in the thoracic limbs in 1 dog, and absent in the right pelvic limb while decreased in left pelvic limb and right thoracic limb in 1 dog. One dog had no noticeable proprioceptive deficits. Spinal reflexes were considered abnormal in 17/20 dogs. Four dogs had increased extensor tone in all limbs, 4 dogs had increased extensor tone in the thoracic limbs, 12 dogs had decreased flexor reflex in the thoracic limbs, and 7 dogs had increased patellar reflex (2/7 with clonus). Reflexes were considered within normal limits in 3 dogs.

Based on spinal reflexes and evidence of pain upon palpation of the neck, neurolocalization for the lesions was C1‐C5 in 8 dogs, C6‐C8 in 3 dogs, and C6‐T2 spinal cord segments in 9 dogs.

Ancillary tests included CBC and biochemistry panel in 14 dogs, packed cell volume (PCV) and total protein (TP) concentration in 6 dogs, cervical spinal radiographs in 10 dogs, cerebrospinal fluid (CSF) analysis in 7 dogs, and computed tomography (CT) in 3 dogs. Overall, CBC, biochemistry panel, PCV, TP, and CSF analysis were within normal limits. Minor changes seen on biochemistry panels were attributed to bone growth in young dogs (increased serum phosphorous concentration and ALP activity in 1 dog and increased serum phosphorous concentration in 1 dog), mild dehydration (increased TP in 1 dog), and corticosteroid administration (increased ALP activity in 1 dog). When available, radiographs showed evidence of osteoarthritic changes characterized by enlarged articular processes with irregular and sclerotic margins (8 dogs), narrowed vertebral canal (1 dog), and narrowed intervertebral disk space (1 dog). CT findings were in accordance with changes seen on MRI, with osseous changes in the articular processes and presumptive sites of spinal cord compression observed in all 3 dogs, vertebral canal stenosis in 1 dog, and marked intervertebral foraminal stenosis in 1 dog.

**Figure 1:**
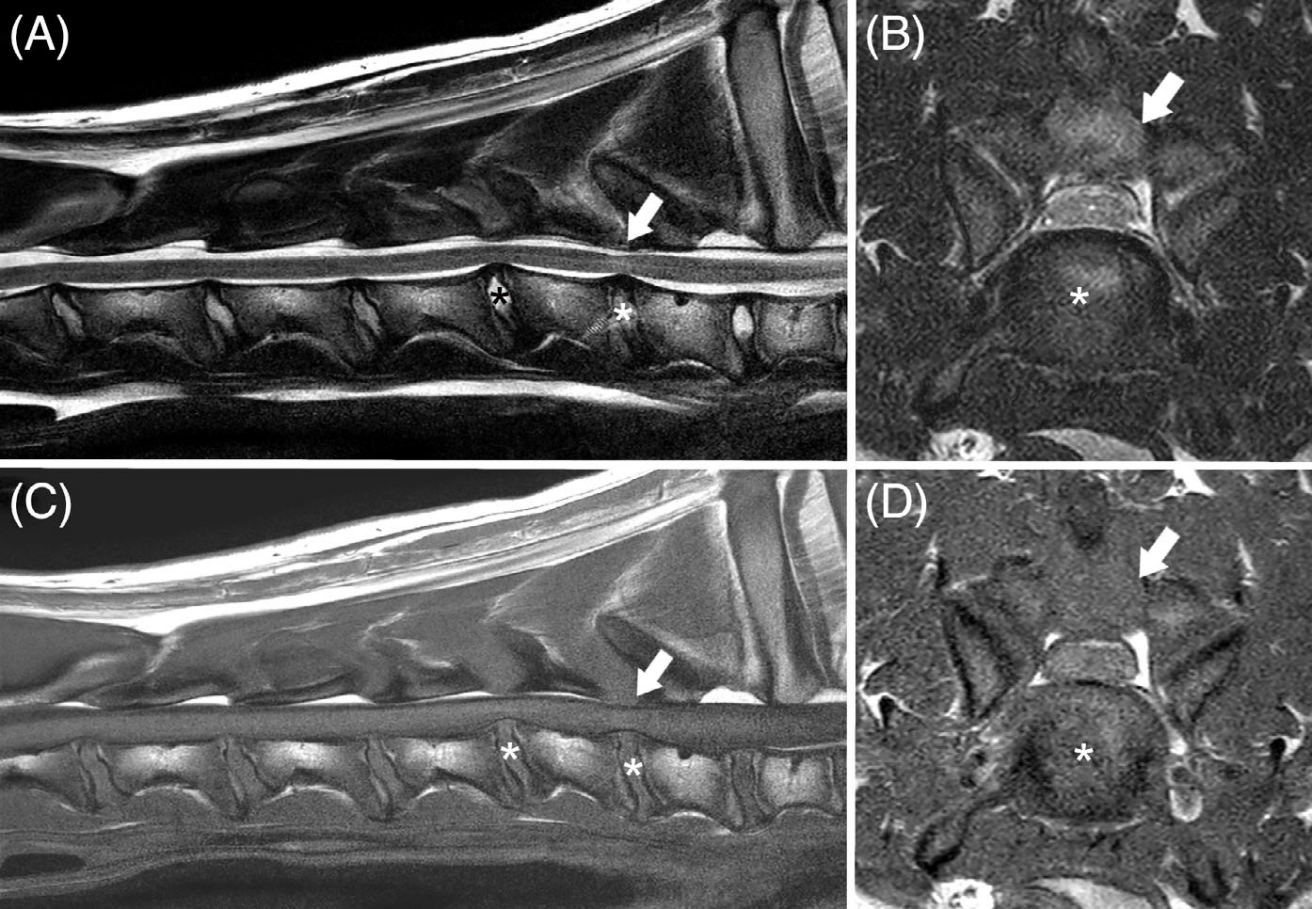
T2‐weighted sagittal (A) and transverse (B) and T1‐weighted sagittal (C) and transverse (D) magnetic resonance imaging of a 6‐month‐old Great Dane with cervical spondylomyelopathy because of intervertebral disk protrusion (asterisks) at C5‐C6 and C6‐C7 and dorsal soft tissue proliferation (arrow) with mild C6 dorsal lamina thickening at C6‐C7

**Figure 2:**
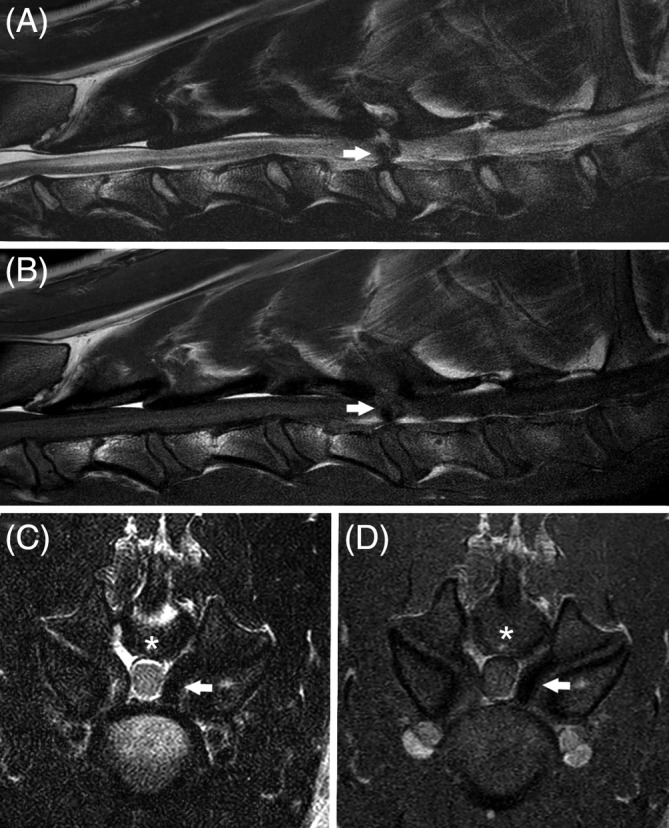
Parasagittal T2‐weighted (A) and T1‐weighted (B), transverse T2‐weighted (C) and T1‐weighted (D) magnetic resonance imaging of a 12‐month‐old Great Dane with cervical spondylomyelopathy because of osseous proliferation of the articular processes, more prominent on the left (arrows). Also, note the hypertrophy of the ligamentum flavum (asterisk)

**Figure 3:**
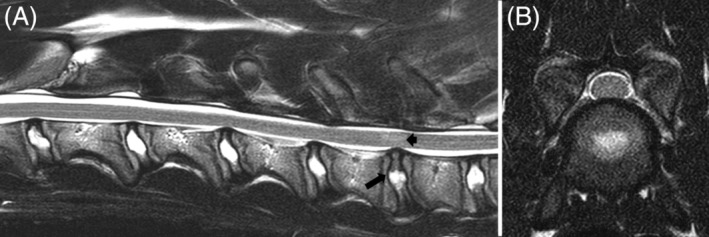
Magnetic resonance imaging of a 7‐month‐old Great Dane with cervical spondylomyelopathy because of intervertebral disk protrusion. A, Sagittal T2‐weighted image showing spinal cord compression at C6‐C7 because of intervertebral disk protrusion (long arrow). Note the partial degeneration of intervertebral disks at C2‐C3, C5‐C6, C6‐C7, C7‐T1. There is also hyperintensity of the spinal cord parenchyma at C6‐C7 (short arrow). B, Transverse T2‐weighted image showing intervertebral disk protrusion causing mild spinal cord compression at C6‐C7

On MRI, the main compression site was located at C5‐C6 in 10 dogs, C6‐C7 in 6 dogs, C4‐C5 in 2 dogs, C3‐C4 in 2 dogs, and C2‐C3 in 2 dogs. In 2 dogs, the main compression site was impossible to distinguish between C5‐C6 and C6‐C7, and both sites were counted as the main site of compression. Twelve dogs had ≥2 sites of spinal cord compression (Figure 1): 5 dogs had 2 sites of compression (C5‐C6 and C6‐C7 in 3/5; C3‐C4 and C6‐C7 in 1/5; C4‐C5 and C6‐C7 in 1/5), 4 dogs had 3 sites of compression (C4‐C5, C5‐C6, and C6‐C7 in 3/4; C3‐C4, C4‐C5, and C5‐C6 in 1/4) and 2 dogs had 4 sites of compression (C2‐C3, C3‐C4, C5‐C6, C6‐C7 in 1/2; C3‐C4, C4‐C5, C5‐C6, C6‐C7 in 1/2), and 1 dog had 5 sites of compression (from C2‐C3 to C6‐C7). Overall, the lesions were classified as primarily osseous‐associated in 18 dogs (Figure 2), whereas 2 had disk protrusion as the main cause of spinal cord compression (1 Doberman Pinscher and 1 Great Dane; Figure 3). One 10‐month‐old Doberman with primarily osseous‐related compression also had spinal cord compression because of intervertebral disk protrusion.

The main cause (and direction) of spinal cord compression was articular process proliferation in 8 dogs (bilateral in 3/8, dorsolateral on both sides in 3/8, ventrolateral on both sides in 1/8, unilateral in 1/8 dog), thickening of the dorsal lamina (dorsal) in 4 dogs, combined hypertrophy of the ligamentum flavum and thickening of the dorsal lamina (dorsal) in 2 dogs, hypertrophy of the ligamentum flavum and “tipping” of the vertebral body (dorsal and ventral) in 2 dogs, intervertebral disk protrusion (ventral) in 2 dogs, combined hypertrophy of the ligamentum flavum and proliferation of the articular processes (dorsal and bilateral) in 1 dog, and hypertrophy of the ligamentum flavum (dorsal) in 1 dog.

Signs of degenerative changes in the articular process joints were observed in all dogs. All dogs had signs of decreased amount of synovial joint fluid in at least 1 location: 8/20 dogs at C2‐C3, 14/20 at C3‐C4, 16/20 at C4‐C5, 17/20 at C5‐C6, and 11/20 at C6‐C7. Synovial joint fluid signal was considered to be completely absent in 2/20 dogs at C2‐C3, 1/20 dogs at C3‐C4, 1/20 dogs at C4‐C5, and in 3/20 dogs at C6‐C7. As for characterization of the regularity of the articular surface and presence of subchondral bone sclerosis, the articular process joint was considered to be smooth with evidence of subchondral bone sclerosis in 6/20 dogs at C2‐C3, 10/20 dogs at C3‐C4, 5/20 dogs at C4‐C5, 3/20 dogs at C5‐C6, and 7/20 dogs at C6‐C7. The articular process joint surface was considered to be irregular with presence of subchondral bone sclerosis in 6/20 dogs at C2‐C3, 6/20 dogs at C3‐C4, 11/20 dogs at C4‐C5, 10/20 dogs at C5‐C6, and 8/20 dogs at C6‐C7.

Nine of 20 (45%) dogs had spinal cord signal changes with hyperintensity on T2W images, but none had hypointensity seen on T1W images. Intervertebral foraminal stenosis was present in 17/20 dogs (85%) to various extents. Nine of 20 dogs (45%) had at least partial degeneration of ≥1 intervertebral disks.

Regarding treatment, exercise restriction was recommended for all dogs with confinement in a large cage or small room recommended for 3 dogs (2 dogs treated surgically and 1 dog treated conservatively).

Follow‐up information was obtained for 12/20 dogs. Follow‐up times ranged from 1 month to 5 years (mean, 1.4 years; median, 1.1 year). The mean and median neurologic scores for these 12 dogs were 2.8 and 3, respectively. Four dogs (4/12) were reportedly stable without long‐term anti‐inflammatory or analgesic medications at 3 months (grade 2; received gabapentin at a dosage of 10 mg/kg q8 hours as needed at the time of diagnosis), 9 months (grade 1), 1.1 year (grade 4; received prednisone at a dosage of 0.32 mg/kg tapered over 3 weeks from the time of diagnosis), and 1.6 years (grade 2). In these dogs, the presenting gait abnormality was ataxia (without paresis), and only 1 dog had neck pain on palpation. One dog (1/12), classified as grade 2, had intermittent waxing and waning of clinical signs over 1 year after diagnosis. At the time of diagnosis, this dog was treated with prednisone at a dosage of 0.5 mg/kg q12h for 10 days, then q24h for 10 days, and then q48h until instructed otherwise. One dog (1/12) on long‐term prednisone treatment was stable at 5 months (grade 3). This dog had been treated with 0.35 mg/kg of prednisone q12h for 7 days, then q24h for 7 days, and decreased to q48h until further notice. Two dogs (2/12) receiving long‐term prednisone were progressively worse 4.5 months and 2 years after diagnosis. The first of these 2 dogs, classified as grade 2, initially received carprofen 2 mg/kg q12h for 15 days, and then was prescribed prednisone at 0.5 mg/kg q12h for 7 days, then q24h for 7 days, and then q48h. The second (grade 4) was prescribed prednisone at 0.6 mg/kg in the morning and 0.30 mg/kg at night for 7 days, followed by 0.6 mg/kg q24h for 7 days, and then q48h. One dog (1/12) was euthanized 1 month after diagnosis (acute onset of tetraparesis with severe proprioceptive ataxia on presentation—grade 4). At diagnosis, this dog had been prescribed 0.6 mg/kg prednisone q12h for 14 days, then q24h for 14 days, as well as 14 mg/kg of gabapentin q8h as needed. Two dogs (2/12), both classified as grade 3, underwent surgical procedures: 1 had cervical dorsal laminectomy and the other had ventral stabilization and dorsal laminectomy. Both reportedly improved after the procedures and still were stable 1.5 and 5 years after surgery, respectively. One dog (1/12), classified as grade 4, died after being attacked by another dog 2 years after diagnosis (before death, the dog had been stable for the first year, but had started to worsen intermittently the last year after a fall). At diagnosis, the dog had been prescribed 0.44 mg/kg prednisone q12h for 7 days, then q24h for 7 days, and then q48h until further notice.

Follow‐up data was not available for 8/20 dogs. The mean and median neurologic scores for these dogs were 2.7 and 2.5, respectively (range, 2‐4). For the 4/8 dogs classified as grade 2, 2/4 were not prescribed long‐term anti‐inflammatory medications, 1/4 was prescribed prednisone (0.42 mg/kg q12h for 14 days, then q24h for 14 days, and then q48h for 4 weeks), and 1/4 was prescribed prednisone (0.43 mg/kg q12h for 10 days, then q24h for 10 days, and then q48h) and gabapentin (10.5 mg/kg q8h for 14 days). Of the 2/8 dogs classified as grade 3, 1 was prescribed prednisone (0.5 mg/kg q12h for 10 days, then q24h for 10 days, and then q48h), and the other was prescribed prednisone (0.44 mg/kg q12h for 14 days, then q24h for 14 days, and then q48h) and tramadol (3.5 mg/kg q 6‐8 h as needed). Of the 2/8 dogs classified as grade 4, 1 was prescribed dexamethasone (0.06 mg/kg q24h for 3 days, then decreased to q48h for 14 days), whereas the other received prednisone (0.34 mg/kg q24h for 14 days, and then q48h for another 14 days).

## 4 DISCUSSION

Juvenile dogs in our study generally had a chronic presentation, with proprioceptive ataxia the most commonly reported gait abnormality, whereas paresis was present in approximately half of the dogs (11/20). Osseous‐associated changes (articular process proliferation, lamina thickening) were the cause of the majority of the spinal cord compressions, although intervertebral disk protrusion and ligamentum flavum hypertrophy also were noted. The most common location of spinal cord compression was C5‐C6, and several dogs had >1 site of compression.

Interestingly, we found disk‐associated CSM in a Doberman and a Great Dane at 5 and 7 months of age, respectively. Disk‐associated CSM typically is seen in large breed dogs with an average age of 7.9 years,^1^ and usually does not develop at such a young age.^6^, ^9^ Also, Great Danes are much more likely to develop osseous‐associated changes rather than disk‐associated changes when younger.^3^, ^5^ Although only 2 dogs had DA‐CSM, it still was surprising to find dogs with primary lesions associated with disk protrusion at such a young age. Very sparse descriptions of this phenomenon were found in the literature.^13^

Also of note was the fact that MRI disclosed changes that were consistent with intervertebral disk degeneration in almost half of the dogs (9/20). This occurrence is much younger than what is usually expected in nonchondrodystrophic breeds, which would be >5 years of age.^29^, ^30^, ^31^, ^32^

Degenerative changes in the articular processes also were frequent, with 19/20 dogs having a decreased amount or absence of synovial joint fluid, signs of subchondral bone sclerosis, or some irregularity in the articular surface in at least 1 site. These observations seem consistent with the osseous proliferative changes observed. For foraminal stenosis, no association was observed between neurologic grade and severity of foraminal stenosis.

Breed distribution for the affected dogs consisted of large and giant breeds, with a higher prevalence of giant breed dogs, primarily Great Danes. This finding was not unexpected, considering the higher prevalence of OA‐CSM in this breed, which has been associated with an earlier onset of clinical signs.^3^, ^5^

Regarding clinical signs on presentation, the most common sign was proprioceptive ataxia, noted in 18 of 20 dogs. Eleven dogs also showed signs of tetraparesis. Most dogs (90%) had a chronic progressive course over a mean of 7.3 weeks. Several owners reported difficulty in recognizing signs of the disease, particularly in distinguishing the normal, clumsy gait of a puppy from an ataxic gait. In a previous report, the mean duration of clinical signs before diagnosis of OA‐CSM in Great Danes was 1.9 years,^33^ thus it is possible that in this report several young adults diagnosed with CSM actually started developing signs when only a few months old. In agreement with findings previously reported for older dogs,^1^ cervical hyperesthesia was observed in 45% of dogs (9/20) but was not the main reason for presentation in any of the dogs.

Regarding the location of the main spinal cord compression, it was consistent with what has been reported previously in the literature,^6^, ^33^ with the majority of lesions affecting the caudal cervical vertebral column. More than half (60%) of the dogs had multiple sites of spinal cord compression, which is not uncommon with CSM.^3^

It can be hypothesized that juvenile dogs with CSM had a more severe manifestation of CSM, causing the disease to manifest clinical signs earlier, and that is why they were diagnosed with the disease at such young age. Regarding treatment, most dogs were managed medically, with outcomes varying from stable to progressive decline. The 2 surgically treated dogs were stable 1.5 and 5 years after surgery. Overall, the presenting neurological signs in the 4 dogs that did not receive long‐term corticosteroid treatment suggest that a milder presentation was the reason these dogs were stable on follow‐up (the dog with the more severe presentation—grade 4—received 3 weeks of prednisone). However, it is difficult to draw conclusions based on the small number of dogs. Dogs with OA‐CSM have been reported to survive several years, whether treated surgically or medically, and improvement is reportedly higher with surgery.^3^ Our own experience suggests that short‐term outcome usually is positive with medical management, whereas long‐term outcome is more variable.

Almost half of the dogs had T2W hyperintensity on MRI. This has been suggested to be associated with a worse prognosis, especially if combined with T1W hypointensity,^34^, ^35^ which was not observed in any of the 20 dogs. The proportion of dogs with T2W hyperintensity (45%) is lower than previously described.^3^, ^6^, ^10^, ^36^ In humans, T2W hyperintensity with T1W isointensity is associated with changes such as edema, gliosis, Wallerian degeneration, demyelination, and some loss of nerve cells.^37^ In dogs, the only experimental study performed suggested a predominance of gray matter changes (motor neuron loss and necrosis).^38^ However, if these signal changes in the spinal cord parenchyma are associated with chronicity,^39^ perhaps these dogs did not have enough time to develop all of the observable lesions on MRI.

One of the limitations of our study was that follow‐up was not available long‐term for all 20 dogs, but this limitation is expected in a retrospective study ranging over a decade. A limitation related to the MRI examinations was that different protocols were used. However, a high‐field MRI was used in all cases, with at least 3 transverse T1‐ and T2‐weighted slices obtained at the 5 cervical intervertebral disk levels (1 transverse slice at the caudal endplate of the cranial vertebra, 1 at the intervertebral disk, and 1 at the cranial endplate of the caudal vertebra).

Another limitation is the relatively small sample size, although the 20 juvenile dogs described here far exceed the previous sporadic reports of CSM in juvenile dogs.

Over the past 45 years, 18 of 52 papers reviewed reported dogs ≤1 year of age in their populations, but most of these did not report the exact age of all dogs or how many dogs were each age range.^3^, ^4^, ^6^, ^10^, ^13^, ^14^, ^15^, ^16^, ^17^, ^18^, ^19^, ^20^, ^21^, ^22^, ^23^, ^24^, ^25^, ^26^ Most studies also did not provide specific clinical or imaging information for each dog, thus making it difficult to analyze the specific behavior of this disease in juvenile dogs.

There are only 2 reports with a larger proportion of juvenile dogs confirmed to have CSM with myelography, CT, or MRI. An MRI study on giant breed dogs with CSM had a large number of young dogs, with 61% of dogs being ≤2 years of age, although it was not specified how many of the dogs were ≤ 1 year of age.^4^ A radiographic and myelographic study from South Africa, specifically on the Boerboel breed with CSM, found a higher proportion (70%) of juvenile dogs with CSM.^23^ It is possible this is a reflection of the large number of Boerboels in that region, and also of the presentation of the disease in that breed.

## 5 CONCLUSIONS

Cervical spondylomyelopathy in juvenile dogs generally was characterized by a chronic presentation, mostly affecting giant breeds, with proprioceptive ataxia with or without tetraparesis as the most common gait abnormality. Compression of the spinal cord occurred most frequently at the caudal cervical vertebral region because of osseous‐associated changes. It was not uncommon for dogs to have >1 site of compression. Compressive lesions caused by intervertebral disk protrusion should not be automatically ruled out because of the young age or breed of a dog, because they were noted in 2 dogs in our study and almost half of the population had intervertebral disk degeneration. Spinal cord parenchyma signal changes were not predominant within the group, and only T2W hyperintensity was observed. Outcome varied greatly, including stable without long‐term corticosteroid administration, stable with surgery, waxing and waning of signs or progressive worsening despite long‐term corticosteroid administration.

## CONFLICT OF INTEREST DECLARATION

Authors declare no conflict of interest.

## OFF‐LABEL ANTIMICROBIAL DECLARATION

Authors declare no off‐label use of antimicrobials.

## INSTITUTIONAL ANIMAL CARE AND USE COMMITTEE (IACUC) OR OTHER APPROVAL DECLARATION

This is a retrospective study. For dogs prospectively enrolled in past studies, all procedures were performed with the approval of the Clinical Research Advisory Committee and the IACUC of The Ohio State University.

## HUMAN ETHICS APPROVAL DECLARATION

Authors declare human ethics approval was not needed for this study.

## References

[1] da Costa RC . Cervical spondylomyelopathy (wobbler syndrome) in dogs. Vet Clin North Am Small Anim Pract. 2010;40:881‐913. 20732597 10.1016/j.cvsm.2010.06.003

[2] De Decker S , da Costa RC , Volk HA , Van Ham LM . Current insights and controversies in the pathogenesis and diagnosis of disc‐associated cervical spondylomyelopathy in dogs. Vet Rec. 2012;171:531‐537. 23180710 10.1136/vr.e7952

[3] Gasper JA , Rylander H , Stenglein JL , et al. Osseous‐associated cervical spondylomyelopathy in dogs: 27 cases (2000‐2012). J Am Vet Med Assoc. 2014;244:1309‐1318. 24846432 10.2460/javma.244.11.1309

[4] Lipsitz D , Levitski RE , Chauvet AE , Berry WL . Magnetic resonance imaging features of cervical stenotic myelopathy in 21 dogs. Vet Radiol Ultrasound. 2001;42:20‐27. 11245233 10.1111/j.1740-8261.2001.tb00899.x

[5] Murthy VD , Gaitero L , Monteith G . Clinical and magnetic resonance imaging (MRI) findings in 26 dogs with canine osseous‐associated cervical spondylomyelopathy. Can Vet J. 2014;55:169‐174. 24489397 PMC3894878

[6] da Costa RC , Parent JM , Holmberg DL , Sinclair D , Monteith G . Outcome of medical and surgical treatment in dogs with cervical spondylomyelopathy: 104 cases (1988‐2004). J Am Vet Med Assoc. 2008;233:1284‐1290. 18922055 10.2460/javma.233.8.1284

[7] De Decker S , Gielen IM , Duchateau L , et al. Morphometric dimensions of the caudal cervical vertebral column in clinically normal Doberman pinschers, English foxhounds and Doberman pinschers with clinical signs of disk‐associated cervical spondylomyelopathy. Vet J. 2012;191:52‐57. 21257325 10.1016/j.tvjl.2010.12.017

[8] da Costa RC , Parent JM , Partlow G , Dobson H , Holmberg DL , LaMarre J . Morphologic and morphometric magnetic resonance imaging features of Doberman pinschers with and without clinical signs of cervical spondylomyelopathy. Am J Vet Res. 2006;67:1601‐1612. 16948609 10.2460/ajvr.67.9.1601

[9] De Decker S , Bhatti SF , Duchateau L , et al. Clinical evaluation of 51 dogs treated conservatively for disc‐associated wobbler syndrome. J Small Anim Pract. 2009;50:136‐142. 19261084 10.1111/j.1748-5827.2008.00705.x

[10] Gutierrez‐Quintana R , Penderis J . MRI features of cervical articular process degenerative joint disease in great Dane dogs with cervical spondylomyelopathy. Vet Radiol Ultrasound. 2012;53:304‐311. 22236021 10.1111/j.1740-8261.2011.01912.x

[11] Martin‐Vaquero P , da Costa RC . Magnetic resonance imaging features of great Danes with and without clinical signs of cervical spondylomyelopathy. J Am Vet Med Assoc. 2014;245:393‐400. 25075822 10.2460/javma.245.4.393 PMC4213553

[12] Provencher M , Habing A , Moore SA , Cook L , Phillips G , da Costa RC . Evaluation of osseous‐associated cervical spondylomyelopathy in dogs using kinematic magnetic resonance imaging. Vet Radiol Ultrasound. 2017;58:411‐421. 28402031 10.1111/vru.12495

[13] Solano MA , Fitzpatrick N , Bertran J . Cervical distraction‐stabilization using an intervertebral spacer screw and string‐of pearl (SOP™) plates in 16 dogs with disc‐associated wobbler syndrome. Vet Surg. 2015;44:627‐641. 25929590 10.1111/vsu.12325

[14] Mason TA . Cervical vertebral instability (wobbler syndrome) in the Doberman. Aust Vet J. 1977;53:440‐445. 588180 10.1111/j.1751-0813.1977.tb05494.x

[15] Denny HR , Gibbs C , Gaskell CJ . Cervical spondylopathy in the dog‐‐a review of thirty‐five cases. J Small Anim Pract. 1977;18:117‐132. 853735 10.1111/j.1748-5827.1977.tb05862.x

[16] Read RA , Robins GM , Carlisle CH . Caudal cervical spondylo‐myelopathy (wobbler syndrome) in the dog: a review of thirty cases. J Small Anim Pract. 1983;24:605‐621.

[17] McKee WM . Dorsal laminar elevation as a treatment for cervical vertebral canal stenosis in the dog. J Small Anim Pract. 1988;29:95‐103.

[18] Lewis DG . Cervical spondylomyelopathy (‘wobbler’ syndrome) in the dog: a study based on 224 cases. J Small Anim Pract. 1989;30:657‐665.

[19] McKee WM , Lavelle RB , Mason TA . Vertebral stabilisation for cervical spondylopathy using a screw and washer technique. J Small Anim Pract. 1989;30:337‐342.

[20] Bruecker KA , Seim HB , Withrow SJ . Clinical evaluation of three surgical methods for treatment of caudal cervical spondylomyelopathy of dogs. Vet Surg. 1989;18:197‐203. 2773281 10.1111/j.1532-950x.1989.tb01070.x

[21] Sharp NJ , Cofone M , Robertson ID , et al. Computed tomography in the evaluation of caudal cervical spondylomyelopathy of the Doberman pinscher. Vet Radiol & Ultrasound. 1995;36:100‐108.

[22] De Risio L , Muñana K , Murray M , Olby N , Sharp NJ , Cuddon P . Dorsal laminectomy for caudal cervical spondylomyelopathy: postoperative recovery and long‐term follow‐up in 20 dogs. Vet Surg. 2002;31:418‐427. 12209412 10.1053/jvet.2002.34673

[23] Gray MJ , Kirberger RM , Spotswood TC . Cervical spondylomyelopathy (wobbler syndrome) in the Boerboel. J S Afr Vet Assoc. 2003;74:104‐110. 15038422 10.4102/jsava.v74i4.520

[24] da Costa RC , Echandi RL , Beauchamp D . Computed tomography myelographic findings in dogs with cervical spondylomyelopathy. Vet Radiol Ultrasound. 2012;53:64‐70. 22093094 10.1111/j.1740-8261.2011.01869.x

[25] Taylor‐Brown FE , Cardy TJ , Liebel FX , et al. Risk factors for early post‐operative neurological deterioration in dogs undergoing a cervical dorsal laminectomy or hemilaminectomy: 100 cases (2002‐2014). Vet J. 2015;206:327‐331. 26542365 10.1016/j.tvjl.2015.10.010

[26] Cooper C , Gutierrez‐Quintana R , Penderis J , Gonçalves R . Osseous associated cervical spondylomyelopathy at the C2‐C3 articular facet joint in 11 dogs. Vet Rec. 2015;177:522. 26510824 10.1136/vr.103104

[27] Costa, 2006 , da Costa RC , Poma R , Parent JM , Partlow G , Monteith G . Correlation of motor evoked potentials with magnetic resonance imaging and neurologic findings in Doberman pinschers with and without signs of cervical spondylomyelopathy. Am J Vet Res. 2006 Sep;67(9):1613‐1620. 16948610 10.2460/ajvr.67.9.1613

[28] De Decker S , Gomes SA , Packer RM , et al. Evaluation of magnetic resonance imaging guidelines for differentiation between thoracolumbar intervertebral disk extrusions and intervertebral disk protrusions in dogs. Vet Radiol Ultrasound. 2016;57:526‐533. 27374979 10.1111/vru.12394

[29] Smolders LA , Bergknut N , Grinwis GC , et al. Intervertebral disc degeneration in the dog. Part 2: chondrodystrophic and non‐chondrodystrophic breeds. Vet J. 2013;195:292‐299. 23154070 10.1016/j.tvjl.2012.10.011

[30] Brisson BA . Intervertebral disc disease in dogs. Vet Clin North Am Small Anim Pract. 2010;40:829‐858. 20732594 10.1016/j.cvsm.2010.06.001

[31] Bach FC , Willems N , Penning LC , Ito K , Meij BP , Tryfonidou MA . Potential regenerative treatment strategies for intervertebral disc degeneration in dogs. BMC Vet Res. 2014;10:3. 24387033 10.1186/1746-6148-10-3 PMC3914844

[32] Smolders LA , Meij BP , Onis D , et al. Gene expression profiling of early intervertebral disc degeneration reveals a down‐regulation of canonical Wnt signaling and caveolin‐1 expression: implications for development of regenerative strategies. Arthritis Res Ther. 2013;15:R23. 23360510 10.1186/ar4157 PMC3672710

[33] Martin‐Vaquero P , da Costa RC , Lima CG . Cervical spondylomyelopathy in great Danes: a magnetic resonance imaging morphometric study. Vet J. 2014;201:64‐71. 24888675 10.1016/j.tvjl.2014.04.011 PMC4169205

[34] Nouri A , Martin AR , Kato S , Reihani‐Kermani H , Riehm LE , Fehlings MG . The relationship between mri signal intensity changes, clinical presentation, and surgical outcome in degenerative cervical myelopathy: analysis of a global cohort. Spine (Phila pa 1976). 2017;42:1851‐1858. 28498290 10.1097/BRS.0000000000002234

[35] Vedantam A , Jonathan A , Rajshekhar V . Association of magnetic resonance imaging signal changes and outcome prediction after surgery for cervical spondylotic myelopathy. J Neurosurg Spine. 2011;15:660‐666. 21923236 10.3171/2011.8.SPINE11452

[36] Eagleson JS , Diaz J , Platt SR , et al. Cervical vertebral malformation‐malarticulation syndrome in the Bernese mountain dog: clinical and magnetic resonance imaging features. J Small Anim Pract. 2009;50:186‐193. 19320813 10.1111/j.1748-5827.2009.00731.x

[37] Ohshio I , Hatayama A , Kaneda K , Takahara M , Nagashima K . Correlation between histopathologic features and magnetic resonance images of spinal cord lesions. Spine (Phila pa 1976). 1993;18:1140‐1149. 8362319 10.1097/00007632-199307000-00005

[38] al‐Mefty O , Harkey HL , Marawi I , et al. Experimental chronic compressive cervical myelopathy. J Neurosurg. 1993;79:550‐561. 8410225 10.3171/jns.1993.79.4.0550

[39] Suri A , Chabbra RP , Mehta VS , Gaikwad S , Pandey RM . Effect of intramedullary signal changes on the surgical outcome of patients with cervical spondylotic myelopathy. Spine J. 2003;3:33‐45. 14589243 10.1016/s1529-9430(02)00448-5

